# Prognostic Factors for Survival at 6-Month Follow-up of Hospitalized Patients with Decompensated Congestive Heart Failure

**Published:** 2010

**Authors:** Mostafa Cheraghi, Masoumeh Sadeghi, Nizal Sarrafzadegan, Ali Pourmoghadas, Mohammad Arash Ramezani

**Affiliations:** 1 Cardiology Fellow, Isfahan University of Medical Science, Isfahan, Iran; 2 Associate Professor of Cardiology, Isfahan Cardiovascular Research Center, Isfahan University of Medical Science, Isfahan, Iran; 3 Professor of Cardiology, Isfahan Cardiovascular Research Center, Isfahan University of Medical Sciences, Isfahan, Iran; 4 Associate Professor of Cardiology, Isfahan University of Medical Science, Isfahan, Iran; 5 Community Medicine Specialist, Isfahan Cardiovascular Research Center, Isfahan University of Medical Science, Isfahan, Iran

**Keywords:** Heart failure, Survival, Mortality

## Abstract

**BACKGROUND:**

The prevalence of Congestive Heart Failure (CHF) is increasing in recent years. Factors associated with mortality in CHF patients are important to be determined in order to select therapeutic modality by physicians. The purpose of the current study was to declare predictors of 6-months survival in patients hospitalized for decompensated CHF in Isfahan.

**METHODS:**

A cohort of 301 hospitalized patients with decompensated CHF were recruited in this study. The diagnosis of CHF was based on previous hospitalizations and Framingham criteria for heart failure (HF). Information regarding past history, accompanying diseases such as cerebrovascular accidents (CVA), chronic obstructive pulmonary diseases (COPD), clinical data, medications and echocardiography were obtained by a cardiologist. Patients were followed for their survival for 6 months by telephone calls. Kaplan-Meier method was used for uni variate survival analysis and Cox proportional hazard model was used for multivariate analysis.

**RESULTS:**

Mean age of patients was 71.9 ± 12.2 years and 59.8% was male. During 6-months follow-up 138 (45.8%) patients died. Mean survival was 119.2 ± 4.4 days (Mean ± SEM). Significant prognostic factors for 6 months survival were high education level (HR = 0.74, CI 95% 0.59—0.93), COPD (HR = 1.91, CI 95% 1.2—3.04), CVA (HR = 1.69, CI 95% 1.03—2.78), Angiotensin Converting enzyme (ACE) inhibitors use (HR = 0.44, CI 95% 0.3—0.66) and Diuretics (HR = 0.63, CI 95% 0.41-0.96).

**CONCLUSION:**

Six-month survival of hospitalized decompensated CHF patients in Iran is not favorable. Many factors particularly accompanying diseases and medications affected the patient's 6-months survival.

## Introduction

One of the important cardiac diseases with poor prognosis is congestive heart failure (CHF). In recent years it has become one of the most important public health problems in cardiovascular medicine.^1^ Despite effective improvement in therapeutics during the past two decades, CHF remains a major cause of cardiovascular morbidity and mortality. Aging of the population and survival improvement of patients with cardiovascular disease (CVD) by modern therapeutic innovations has led to the increasing prevalence of CHF.^2^–^4^

Since 1980, many studies have been reported a progressive improvement in the survival of CHF patients.^5^ However, the average survival remained poor after hospitalization for the first episode of or decompensated CHF.^5^, ^6^ Mortality rate have increased after CHF hospitalization, even after adjustment for baseline predictors of death .^7^ The increased risk of death was highest within one month of discharge and declined progressively over time.^6^

Despite the available data on other CVD, There are few data regarding CHF mortality and morbidity in Iran. Determining predicting factors that one related to mortality and morbidity of hospitalized decompensated CHF patients may help identify which patients need intensive monitoring during hospitalization and after discharge.

So, the aim of this study was to determine the predictors of 6-months survival in patients hospitalized for decompensated CHF in Isfahan.

## Materials and Methods

### Data collection

This cohort included 301 patients, hospitalized for decompensated CHF in two referral hospital for cardiac patients. As there two referral centers sample was representative of the patient's community. The diagnosis was done based on Framingham criteria of CHF by a cardiologist^6^, ^8^. Acute decompensation of CHF was defined by the presence of an acute increase of shortness of breath, pulmonary rales, vascular enlargement and/or frank edema detected by chest X-ray at the time of admission^9^, ^10^. At first a questionnaire was filled including demographics (sex, age, educational level, marital status and smoking (cigarette and /or opium history), medications such as (angiotensin converting enzyme (ACE) inhibitors, diuretics, beta blockers and other drugs). The history of diabetes mellitus (DM), myocardial infarction (MI), chronic obstructive pulmonary disease (COPD), renal disease and hypertension (HTN) were recorded.

Each patient underwent an echocardiography by a cardiologist. A thorough two-dimensional and Doppler echocardiographic study was performed according to a standard imaging protocol. Ejection fraction (EF) was determined. All measurement was done by VIVID 3 echocardiography machine, manufactured in 2006, General Electric Company. EF more or less than 50% was considered normal or low^11^. Blood pressure was taken by standard protocol at the time of echocardiography^12^.

After discharge, patients were followed up for 6 months by telephone calls. Survival status was assessed by telephone contact with family members and verified by studying hospital records. At the end of the follow-up period, the collected data were subjected to statistical analysis.

### Statistical analysis

At the first step, data were described in tables and shown with central and distributional statistical indices. Then, survival analysis was done. Survival curves were plotted and stratified by EF groups using the Kaplan Meier method. The log-rank test was used to test for differences between the survival curves. Cox proportional hazards model was used for multivariate analysis. Statistical analyses were performed via SPSS for Windows, version 15; (SPSS Inc., Chicago, IL). Parametric data are presented as mean±SD or mean±SEM.

P value <0.05 was considered statistically significant.

### Ethical issues

The study was approved by the Ethics Committee in Isfahan Cardiovascular Research Center, WHO- Collaborating Center for Research and Training in Cardiovascular Diseases Control.

## Results

We recruited 301 patients aged 19-92 years old,with decompensated CHF who were admitted to the cardiology departments in two referral hospital. The baseline characteristics of studied patients have been shown in table 1. Mean age of studied patients was 71.9±12.2.

**Table 1:** Baseline characteristics of CHF patients

Variable		Frequency	%
Sex	Male	*180*	*59.8*
	Female	*121*	*40.2*
Age (years)	<65	*80*	*26.6*
	≥65	*221*	*73.4*
Marital Status	Single	*35*	*11.6*
	Married	*266*	*88.4*
Education	Illiterate	*115*	*38.2*
	Primary school	*94*	*31.2*
	Intermediate school	*3*	*1*
	Diploma	*81*	*26.9*
	Above diploma	*8*	*2.7*
Current Smoker	No	*262*	*87*
	Yes	*39*	*13*
Opium Addict	No	*290*	*96.3*
	Yes	*11*	*3.7*
Diabetes Mellitus	No	*192*	*63.8*
	Yes	*109*	*36.2*
Hypertension	No	*162*	*53.8*
	Yes	*139*	*46.2*
Myocardial Infraction	No	*241*	*80.1*
	Yes	*60*	*19.9*
Cerebrovascular Accident	No	*273*	*90.7*
	Yes	*27*	*9.3*
COPD*	No	*264*	*87.7*
	Yes	*37*	*12.3*
Renal Diseases	No	*273*	*90.7*
	Yes	*28*	*9.3*

COPD* Chronic Obstrutive Pulmonary Disease

During hospitalization, normal and low EF were seen in 43 (14.3%) and 258 (85.7%) of patients, respectively. Mean level of EF was 29.5%±14.5. Most of patients were on medication and ACE inhibitors and diuretics were the major drugs which were used by patients (Table 2).

**Table 2:** Frequency of medication use in patients

Drug		Number	Frequency %
ACEI*	No	79	26.2
	Yes	222	73.7
Diuretics	No	49	16.3
	Yes	252	83.7
Beta blocker	No	233	77.4
	Yes	68	22.6
Other medication	No	44	14.6
	Yes	257	85.4

ACEI* Angiotensin Converting Enzyme Inhibitors

**Figure 1:**
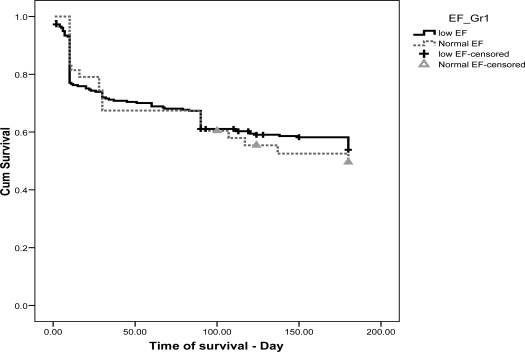
Survival of hospitalized decompensated CHF patients with normal and low EF

During 6-months follow-up 45.8% (138) of patients died. Mean survival was 119.2±4.4 days (Mean±SEM). We compared the survival between patients with normal and low EF. Kaplan-Meier analysis with log rank test did not show any significant differences between two groups (Figure 1).

For determining the different groups of prognostic factors on survival of patients with CHF, three multivariate models of Cox’ regressions were run. The first model was done on socio-demographic variables (Sex, age, education, marital status, and smoking habits). The second model was based on co-morbid diseases like HTN, DM, MI, CVA, COPD, and renal disease. The third hazard model was selected on the drug prescription. The predictive value as hazard ratio were obtained and shown in table 3.

**Table 3:** Independent predictors of 6-months survival of hospitalized decompensated CHF patients

	Predictors	Hazard Ratio	95% CI	P value
Model 1	Sex (female)	*1.03*	*0.72—1.48*	N.S*
	Age ≥65 years	*1.58*	*0.81—3.07*	*N.S*
	Education	*0.74*	*0.59—0.93*	*0.01*
	Marital Status (married)	*0.69*	*0.39—1.22*	*N.S*
	Current smoker	*1.12*	*0.64—1.94*	*N.S*
	Opium addict	*0.71*	*0.30—1.68*	*N.S*
Model 2	Diabetes Mellitus	*1.22*	*0.85—1.78*	*N.S*
	Hypertension	*0.69*	*0.48—1*	*N.S*
	COPD**	*1.91*	*1.20—3.04*	*0.007*
	Myocardial infarction	*1.17*	*0.78—1.76*	*N.S*
	Cerebrovascular accident	*1.69*	*1.03—2.78*	*0.037*
	Renal Disease	*1.13*	*0.65—1.98*	*N.S*
Model 3	ACEI¶	*0.44*	*0.30—0.66*	*<0.001*
	Diuretics	*0.63*	*0.41—0.96*	*0.03*
	Beta Blockers	*0.77*	*0.49—1.21*	*N.S*
	Other Medications	*1.59*	*0.91—2.78*	*N.S*

NS* Non significant

COPD** Chronic Obstrutive Pulmonary Disease

ACEI¶ Angiotensin Converting Enzyme Inhibitors

## Discussion

Our study provides a 6-month follow-up of a cohort of patients with CHF who were admitted in two referral hospitals in Isfahan city. In this study, we present prognostic factors regarding the survival of these patients.

Mortality of CHF during the 6-month follow-up was 45.8%. Previous study reported a mortality rate of 22% during 6-month follow-up in their study in 2005^13^. However, other studies showed different mortality rate of 2% after one year follow-up^14^. Another study from Denmark presented 54% death rate^15^. On the other hand, a study in Spain demonstrated death rate of CHF to be 66.3%^5^. The follow-up period in both studies was 5 years^5^, ^15^. The death rate of decompensated hospitalized CHF patients after 6-months follow-up was very high in our study. It seems that it depends on factors such as medical care and new technology, etiology of CHF, EF level and socioeconomic factors. Moreover, different case selection and definitions, or ethno-racial differences many lead to various results between studies. In our study we recruited hospitalized decompensated CHF patients.

One of prognostic factor on CHF is EF. However, nearly, 14% of our patients had a normal EF and their outcome regarding mortality and morbidity was severe as in patients with reduced EF^16^. Some studies have showed that EF alone is not a predictive factor for CHF Prognosis^17^ whereas other echocardiographic findings like diastolic dysfunction and left ventricular hypertrophy play important roles in prognosis of CHF patients^17^. In our study, there was no significant difference between the survival of CHF patients with normal EF and reduced EF that may be due to other variables that we did not include in the study.

Multivariate analysis in the current study demonstrated that comorbid diseases like COPD and CVA were prognostic factors for mortality in CHF patients. In contrast, high educational level and prescription of ACEIs and diuretics play protective role in the survival of CHF patients.

Although it has been reported that some factors such as comorbid diseases, anemia, some biochemical markers, hyperlipidemia, cardiac function and markers of physical performance affect the survival of CHF patients, 18, but in our study, we could not study all factors. Another reason for the difference between our and other reports is the diversity in the prognosis of CHF, depending on the methods used for diagnostic purposes, the study design, and the underlying diseases. Investigations about CHF have been done in various distinct populations that include outpatients^19^–^21^, inpatients hospitalized for disorders other than CHF^22^, in patients with new onset CHF^23^ or patients hospitalized for worsened CHF.^10^, ^24^–^26^

We had some limitations in our study. We did not assess biochemical markers related to CHF. Furthermore, the underlying disease of CHF of our patients was not determined.

## Conclusion

According to our results, the 6 months prognosis of our hospitalized patients for decompensated CHF was poor. As higher education level had favorable prognostic impact on the survival, actions to improve the patient's awareness and training regarding these diseases may be effective. We also suggest careful investigation and better control of comorbid discovers to have better survival of CHF patients.
